# Prostate microcalcification crystallography as a marker of pathology

**DOI:** 10.1038/s41598-025-98692-8

**Published:** 2025-04-29

**Authors:** Sarah B. Gosling, Emily L. Arnold, Lois Adams, Paul Cool, Kalotina Geraki, Mark O. Kitchen, Iain D. Lyburn, Keith D. Rogers, Tim Snow, Nick Stone, Charlene E. Greenwood

**Affiliations:** 1 School of Chemical and Physical Sciences, Keele University, Keele, Staffordshire, ST5 5BG UK; 2 Diamond Light Source, Harwell Science and Innovation Campus, Didcot, OX11 0DE UK; 3 Robert Jones and Agnes Hunt Orthopaedic Hospital NHS Foundation Trust, Oswestry, Shropshire, SY10 7AG UK; 4 School of Medicine, Keele University, Keele, Staffordshire, ST5 5BG UK; 5 Cranfield Forensic Institute, Cranfield University, Shrivenham, SN6 8LA UK; 6 Thirlestaine Breast Centre, Gloucestershire Hospitals NHS Foundation Trust, Cheltenham, Gloucestershire, GL53 7AS UK; 7 Cobalt Medical Charity, Cheltenham, GL53 7AS UK; 8 Department of Physics and Astronomy, University of Exeter, Exeter, EX4 4QL UK

**Keywords:** Prostate cancer, Calcification, Biomarkers, Prostate cancer, Cancer microenvironment, Biomineralization, Prognostic markers

## Abstract

Prostate cancer remains the most common male cancer; however, treatment regimens remain unclear in some cases due to a lack of agreement in current testing methods. Therefore, there is an increasing need to identify novel biomarkers to better counsel patients about their treatment options. Microcalcifications offer one such avenue of exploration. Microfocus spectroscopy at the i18 beamline at Diamond Light Source was utilised to measure X-ray diffraction and fluorescence maps of calcifications in 10 µm thick formalin fixed paraffin embedded prostate sections. Calcifications predominantly consisted of hydroxyapatite (HAP) and whitlockite (WH). Kendall’s Tau statistics showed weak correlations of ‘a’ and ‘c’ lattice parameters in HAP with GG (r_τ_ = − 0.323, *p* = 3.43 × 10^–4^ and r_τ_ = 0.227, *p* = 0.011 respectively), and a negative correlation of relative zinc levels in soft tissue (r_τ_ = − 0.240, *p* = 0.022) with GG. Negative correlations of the HAP ‘a’ axis (r_τ_ = − 0.284, *p* = 2.17 × 10^–3^) and WH ‘c’ axis (r_τ_ = − 0.543, *p* = 2.83 × 10^–4^) with pathological stage were also demonstrated. Prostate calcification chemistry has been revealed for the first time to correlate with clinical markers, highlighting the potential of calcifications as biomarkers of prostate cancer.

## Introduction

Prostate cancer is the most common cancer in men in the UK, accounting for 27% of all new male cancer cases^1^. Ten-year survival rates are good (78%), despite significant variation in treatments. Many men with high-risk disease are undertreated, particularly older men and those from low socioeconomic backgrounds^2–5^. Similarly, 92–98% of men with low-risk disease are over-treated, for example with radical surgery^2^. This impacts overall patient mortality and quality of life^2–4^. Current diagnostic and prognostic methods also contribute to differences in treatment, where prostate specific antigen (PSA) testing has a high false positive rate (75%) and is incongruent with MRI results in 15–20% cases^3,4^. Given potential undertreatment of high-risk cases, overtreatment of low-risk cases and poor diagnostic test performance, diagnostic and prognostic biomarkers are urgently needed to better inform men of their risks and treatment options and improve clinical outcomes.

One such biomarker for prostate cancer may be calcification chemistry. Calcifications are deposits of calcium salts, primarily consisting of hydroxyapatite (HAP, Ca_10_(PO_4_)_6_(OH)_2_), which have been studied in detail in breast cancer, due to their appearance on mammograms. More recent studies have focussed on exploiting differences in the chemical and crystallographic composition of these deposits to understand the microenvironmental changes and develop novel biomarkers^5,6^. Calcifications in the prostate are less well studied, though some evidence is emerging that prostate calcification may be associated with cancer prognosis^7,8^. Causes of calcifications in the prostate are many-fold, including urinary retention, historic sexually transmitted diseases, inflammatory conditions and prostate cancer, meaning calcifications appear in normal, benign and malignant prostate tissue and vary in size, morphology and composition^9–11^. The exact formation mechanisms of prostate calcification are debated, with both deposition of calcium minerals onto corpora amylacea (amyloid bodies linked to cell degradation) and active deposition by osteoblast-like cells suggested as potential modes of action^7,12^. Previous studies have identified calcification across the prostate, in all four prostatic zones: peripheral (PZ), central (CZ), transition (TZ) and fibromuscular (FMZ)^13^. Some studies have specifically associated PZ calcification with prostate cancer, highlighting the potential of these deposits as biomarkers in the prostate^14^.

In terms of chemistry or crystallographic phase, the presence of hydroxyapatite (HAP), whitlockite (WH, Ca_9_(MgFe)(PO_4_)_6_PO_3_OH), calcium oxalate, in monohydrate and dihydrate forms, brushite, struvite and octacalcium phosphate (OCP) have been reported for prostatic calcifications, although distinctions between cancerous and non-cancerous calcification was not always apparent^7,12,15^. Studies in breast calcifications have linked calcification mineral composition and chemistry to tissue microenvironment, including WH presence and sodium and carbonate levels^5,6,16–18^. Therefore, this study proposes that different microenvironments in normal and malignant prostate tissue, including pH and ion concentrations will impact mineral deposition. For the first time, this novel study investigates prostate calcifications from a crystallographic and elemental perspective, offering an unprecedented insight into prostate tissue microenvironmental nuances at the nanoscale.

## Results

### Calcification crystalline phases

**Fig. 1:**
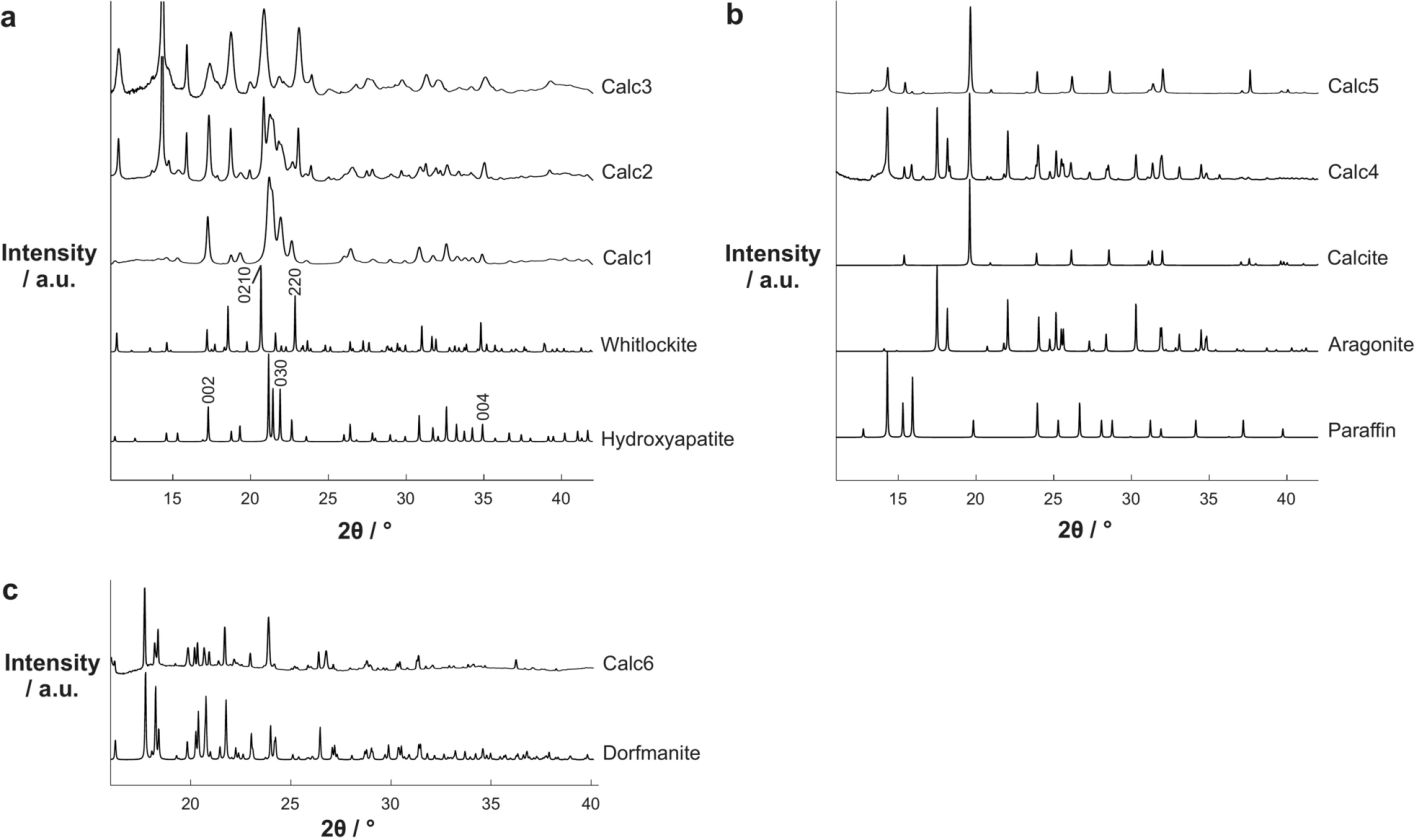
Diffractograms of examples of mineral phases in prostate calcifications. (**a**) Standard patterns for calcium phosphate mineral phases, hydroxyapatite and whitlockite, plus example diffractograms of three calcifications containing only hydroxyapatite (Calc 1), a mixture of hydroxyapatite and whitlockite (Calc 2) and only whitlockite (Calc 3). (**b**) Standard patterns for calcium carbonate mineral phases, aragonite and calcite, and example diffractograms of two calcifications containing calcite and aragonite (Calc 4) and only calcite (Calc 5). A diffractogram of paraffin is also included as this accounts for some of the diffraction peaks observed in the data due to the use of FFPE tissue. (**c**) A standard pattern of dorfmanite plus an example calcification containing this mineral phase (Calc 6).

Several different mineral phases were identified in prostate calcifications, varying dependent on prostatic zones. HAP was identified as the major mineral phase in all zones (Fig. 1a, Calc1). WH was also noted in all zones, both in combination with HAP (Fig. 1a, Calc2) and in isolation (Fig. 1a, Calc3).

In addition, calcium carbonate (CaCO_3_) was identified in two crystallographic forms, namely aragonite and calcite. Aragonite was identified in a single calcification in the peripheral zone within a GG 3 (Gleason 4 + 3) graded tumour, in combination with calcite (Fig. 1b, Calc 4). Calcite was identified in calcifications from all four prostatic zones, to varying extents, both individually and in combination with other mineral phases (Fig. 1b, Calc 4 and 5).

Interestingly, a novel phase, matching dorfmanite (Na_2_(PO_3_OH)•2(H_2_O)), was identified in a single calcification in the peripheral zone of a GG 1 tumour (Fig. 1c).

### Calcifications differ in prostatic zones

**Fig. 2:**
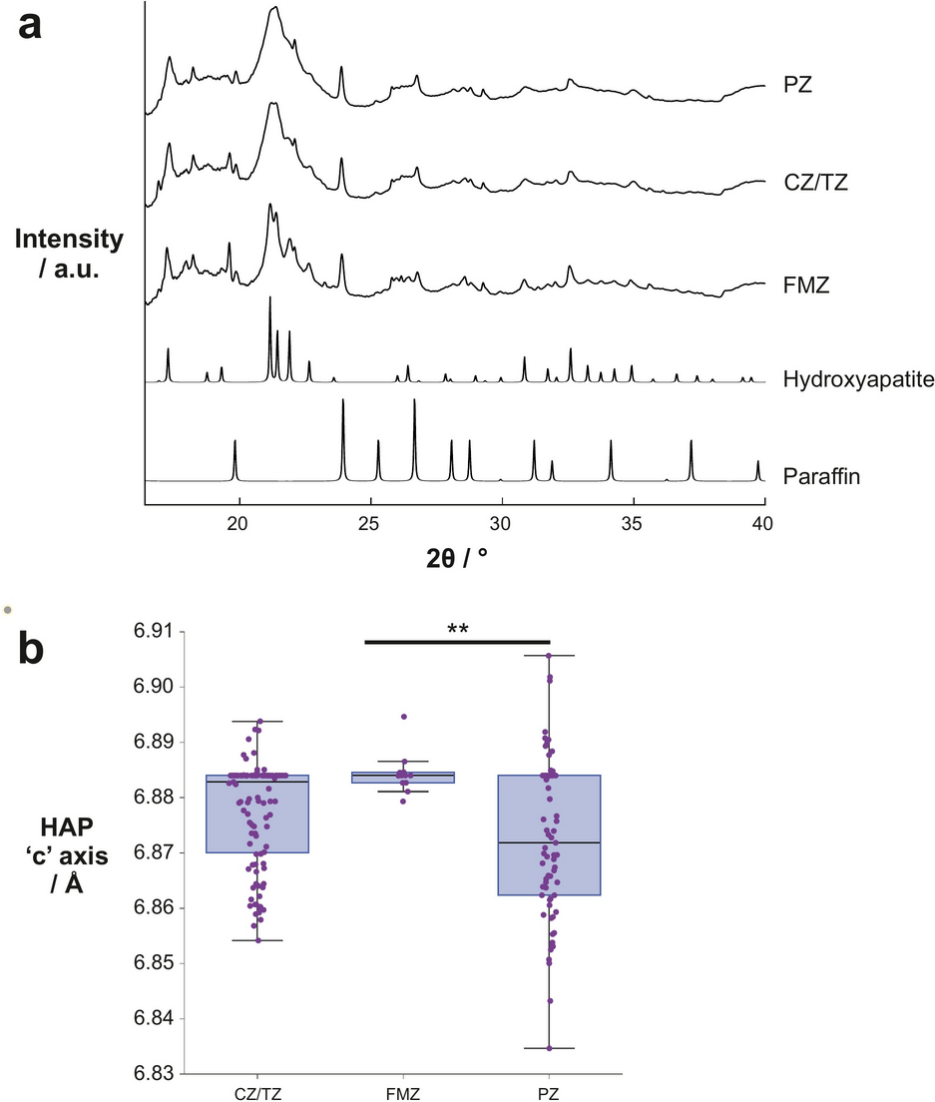
Comparison of calcification crystallography in different prostatic zones. (**a**) Average diffractograms for each prostatic zone, and standard patterns of hydroxyapatite and paraffin. (**b**) Box plot of HAP ‘c’ axis in calcifications in the different prostatic zones. Each point represents the average measurement for an individual calcification. PZ: Peripheral zone, CZ/TZ: Central/Transition zones, FMZ: Fibromuscular zone, HAP: hydroxyapatite. **p < 0.01.

Using median data for each calcification, the principal mineral phases were HAP and WH, with other phases below the limit of detection (4%) (Fig. 2a). Despite an apparent higher crystallinity of FMZ calcifications due to a decrease in peak overlap, there were no significant differences observed in coherence lengths of HAP or WH between the groups (Supplementary Figure S1).

However, a significant difference (*p* = 0.0082) was observed between FMZ and PZ calcifications in the HAP ‘c’ axis (Fig. 2b), though this was not observed in the ‘a’ axis or in WH lattice parameters. Comparing lattice parameters and levels of zinc associated with calcification in each zone, a weak positive correlation was observed between the HAP ‘a’ axis and zinc levels in CZ/TZ (r_τ_ = 0.172, *p* = 0.023) but not in other crystallographic parameters (Supplementary Table S4). Weak negative correlations were also observed between calcification zinc levels and coherence lengths CL 0210 (r_τ_ = − 0.247, *p* = 0.012) and CL 220 (r_τ_ = − 0.231, *p* = 0.019) in WH in PZ calcifications. Further, relative zinc levels in the surrounding soft tissue weakly correlated with the HAP ‘a’ axis (r_τ_ = − 0.240, *p* = 0.022) and whitlockite weight percentage (r_τ_ = − 0.308, *p* = 5.88 × 10^–3^) in PZ calcifications (Supplementary Table S4). It was not possible to make these comparisons for calcifications in the FMZ or in further analyses (grade group and pathological stage) due to low sample numbers per group (n < 30).

### Classification of calcifications by clinical features

Further analysis focussed on calcifications in the peripheral zone, where the majority of prostate cancer is located. Data were first considered as control (adjacent normal tissue from patients with prostate cancer) or malignant, then further divided based on standard clinical features. These included International Society of Urological Pathology (ISUP) Grade (Groups 1–5) and pathological stage, which denotes the size or area of tumour as part of the TNM staging system. These results are outlined below.

#### Calcifications in control versus malignant tissue

Separating data into two groups: control (adjacent normal tissue) or malignant (tumour tissue across a range of grades), no significant differences were apparent between the crystallographic properties (‘a’ and ‘c’ axes or coherence length) of the two groups. Contrarily, relative zinc levels in the calcifications and surrounding tissue were significantly lower in malignant cases (*p* = 0.025 & *p* = 0.017) (Supplementary Figure S3). Numbers in the control group were also relatively low (n = 2), therefore, further analysis only considered malignant data.

#### Calcifications differ between prostate grade groups

**Fig. 3:**
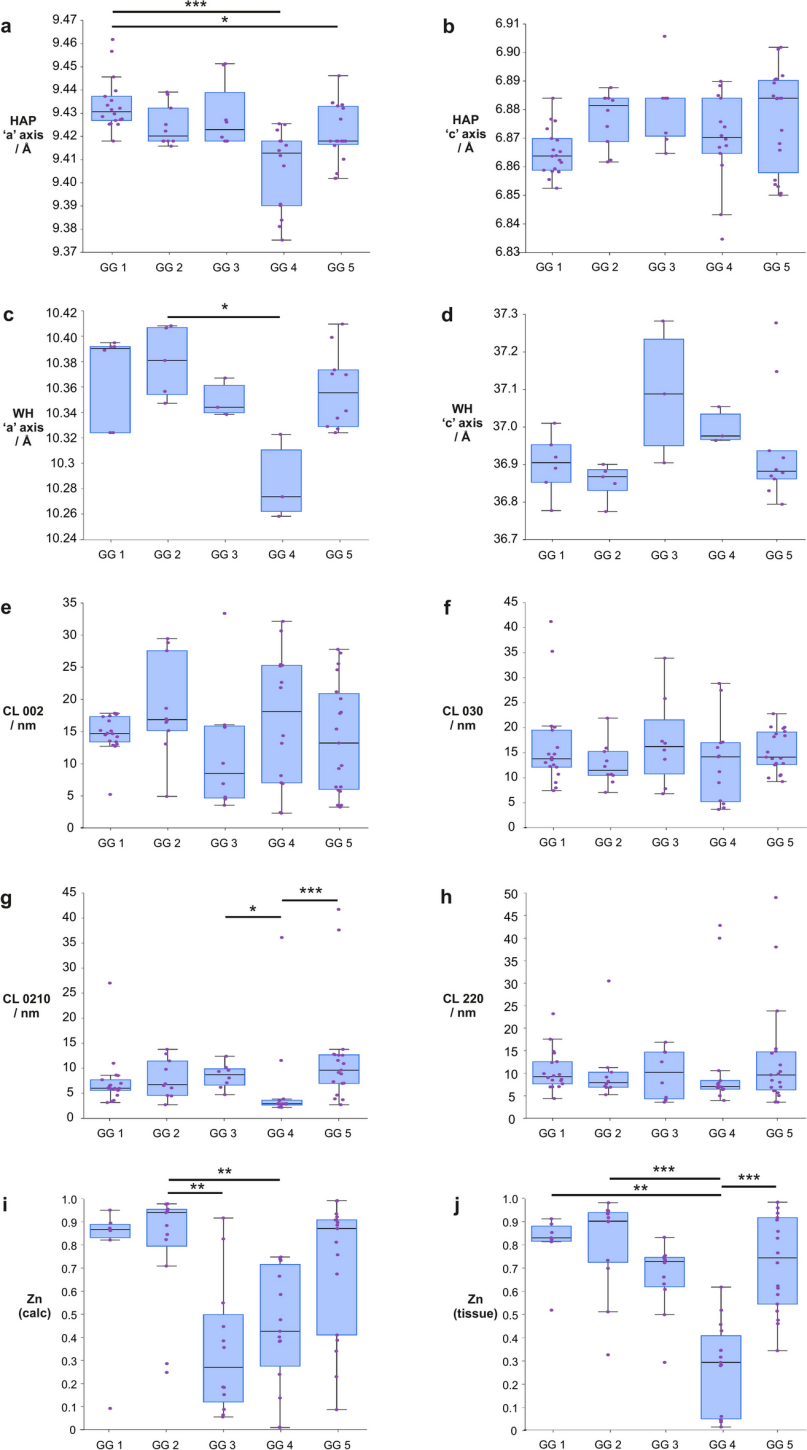
Crystallographic parameters of HAP and WH and zinc distribution averaged by calcification then by grade group (ISUP Grade Groups (GG) 1–5). (**a** and **b**) ‘a’ and ‘c’ axis values for HAP, (**c** and **d**) ‘a’ and ‘c’ axis values for WH. ‘a’ and ‘c’ axes represent physical dimensions of crystal units and can indicate ion substitutions. (**e** and **f**) CL measured along 002 and 030 for HAP, (**g** and **h**) CL measured along 0210 and 220 for WH. (**i** and **j**) Relative zinc levels in prostate calcifications and surrounding soft tissue, measured against total metal ion content (calcium, zinc and iron), presented as a ratio (0–1). Each point represents the average measurement for an individual calcification. HAP: hydroxyapatite, WH: whitlockite, CL: coherence length (a measure of crystallinity in a given direction in a crystal). *p < 0.05, **p < 0.01, ***p < 0.001.

Data from calcifications in malignant tissue were further considered by ISUP Grade (GG 1–5). In HAP, the ‘a’ axis showed a weak negative correlation (r_τ_ = − 0.323, *p* = 3.43 × 10^–4^) with increasing grade group and weak positive correlation (r_τ_ = 0.227, *p* = 0.011) in the ‘c’ axis (Fig. 3a,b). Significant differences were noted between GG 1 and GG 4 and 5 (*p* = 1.1 × 10^–5^ and *p* = 0.045) for the HAP ‘a’ axis (Fig. 3a), but no significant differences were noted for the ‘c’ axis (Fig. 3b).

There were no statistically significant correlations in the ‘a’ or ‘c’ axes of WH with increasing grade group (Fig. 3c,d). GG 2 and GG 4 significantly differed (*p* = 0.036) for WH ‘a’ axis (Fig. 3c), GG 4 had notably lower values for ‘a’ axis length in both HAP and WH, deviating from the apparent trends in the other groups.

Coherence length (CL) was also calculated for several crystallographic peaks for both HAP and WH. For HAP, CL for 002 and 030 were determined, with no significant trends observed between grade groups for these parameters (Fig. 3e,f). There was an overall increasing trend with grade group for CL along 0210 for WH, but no clear trends for 220 (Fig. 3g,h) GG 4 significantly differed from GG 3 (*p* = 0.038) and GG 5 (*p* = 4.8 × 10^–4^) for WH CL0210 (Fig. 3g).

Using XRF analysis, the level of zinc relative to the total metal ion content (calcium, zinc, and iron) was calculated. Relative zinc levels in calcifications were similar for GG 1 and 2, dropping significantly to GG 3, then increasing up to GG 5. Relative zinc levels within calcifications did not significantly correlate with grade group, however significant differences were observed between GG 2 and: GG 3 (*p* = 5.50 × 10^–3^) and GG 2 and GG 4 (*p* = 0.011) (Fig. 3i).

Soft tissue zinc levels showed a weak negative correlation (r_τ_ = − 0.303, *p* = 1.28 × 10^–3^) with increasing grade group, with significant differences noted between GG 4 and: GG 1 (*p* = 3.20 × 10^–3^); GG 2 (*p* = 1.28 × 10^–5^); and GG 5 (*p* = 4.42 × 10^–4^) (Fig. 3j).

#### Calcifications vary by pathological stage

**Fig. 4:**
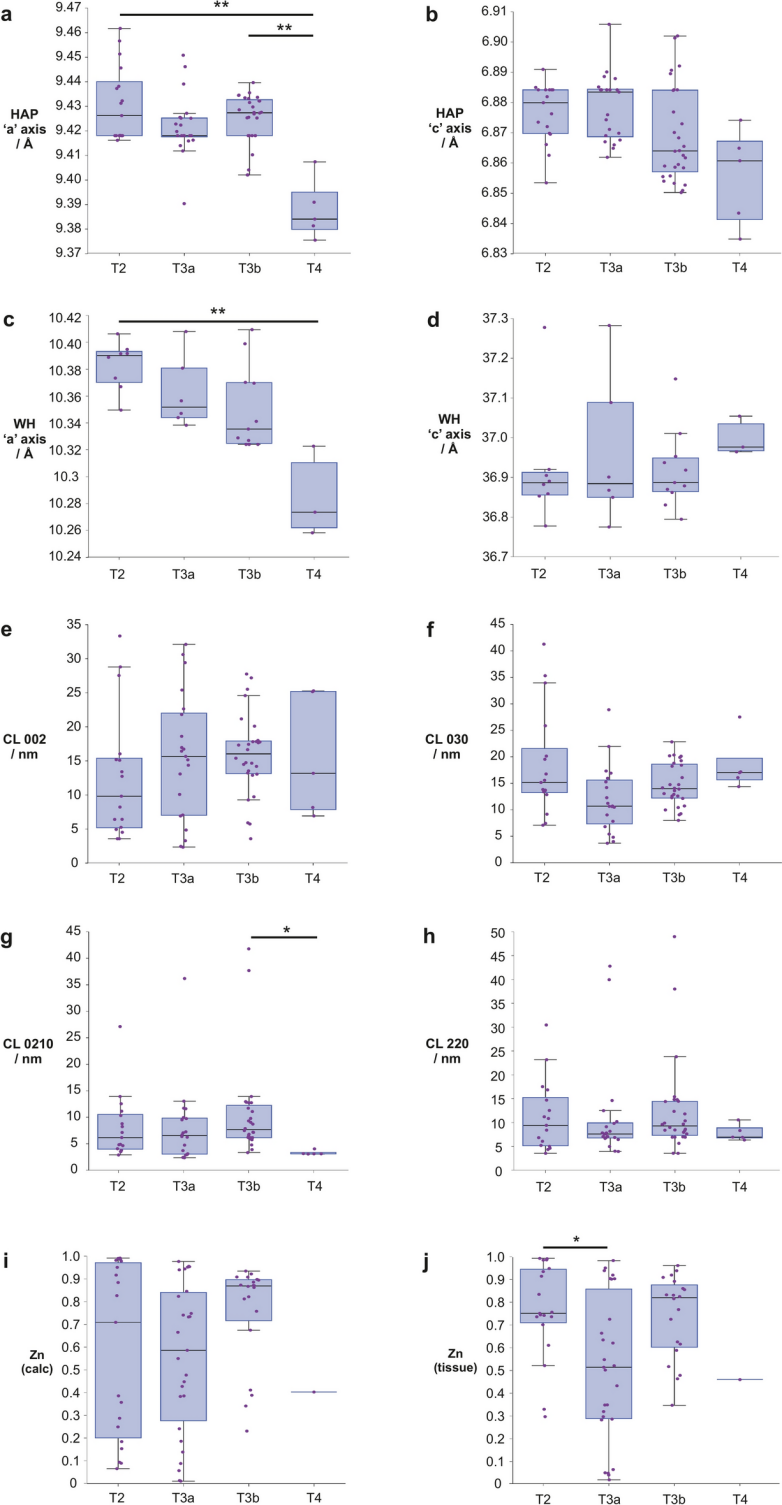
Crystallographic parameters of HAP and WH and zinc distribution averaged by calcification then by pathological stage (size or area of cancer, from inside the prostate gland (T2) to local spread (T4)). (**a** and **b**) ‘a’ and ‘c’ axis values for HAP, (**c** and **d**) ‘a’ and ‘c’ axis values for WH. ‘a’ and ‘c’ axes represent physical dimensions of crystal units, and can indicate ion substitutions. (**e** and **f**) CL measured along 002 and 030 for HAP, (**g** and **h**) CL measured along 0210 and 220 for WH. (**i** and **j**) Relative zinc levels in prostate calcifications and surrounding soft tissue, measured against total metal ion content (calcium, zinc and iron), presented as a ratio (0–1). Each point represents the average measurement for an individual calcification. HAP: hydroxyapatite, WH: whitlockite, CL: coherence length (a measure of crystallinity in a given direction in a crystal). *p < 0.05, **p < 0.01.

Both lattice parameters (‘a’ and ‘c’ axes) for HAP showed a decreasing trend with increasing pathological stage, with a significant weak negative correlation between the ‘c’ axis and pathological stage (r_τ_ = − 0.284, *p* = 2.17 × 10^–3^) (Fig. 4a,b). Significant differences were observed between T4 and T2 (*p* = 1.70 × 10^–3^) and T4 and T3b (*p* = 2.55 × 10^–3^) for the ‘a’ axis. No significant differences were observed for the HAP ‘c’ axis.

A significant moderate negative correlation (r_τ_ = − 0.543, *p* = 2.83 × 10^–4^) was observed in WH ‘a’ axis with increasing pathological stage with a significant difference between T2 and T4 (*p* = 5.60 × 10^–3)^ (Fig. 4c). There was no significant trend or difference between groups for the WH ‘c’ axis (Fig. 4d).

There were no clear trends in CL for HAP or WH (Fig. 4e–h), though T4 was significantly lower (*p* = 0.010) than T3b for WH CL0210 (Fig. 4g).

Zinc levels in both calcifications and the surrounding tissue did not appear to follow clear trends with pathological stage (Fig. 4i,j) though significant differences were observed between T2 and T3a for tissue levels (*p* = 0.022).

## Discussion

Calcifications were identified in 94% of all samples measured, recognising these deposits as a common feature of prostate tissue. HAP and WH are well documented calcium phosphate phases in the human body and the presence of these phases in prostate calcifications is complementary to findings in the breast. Equally, key parameters such as coherence length and lattice parameters were found in a comparable order to those identified in HAP and WH in the breast, highlighting further similarity between these two cancers at a nanoscale^5,6,16,19,20^. However, identification of other mineral phases such as calcite in prostate calcifications highlights the broader phase landscape in prostate compared to breast.

Previous studies have indicated the importance of ion presence and pH in the control of HAP and WH formation. Elements such as zinc and magnesium can influence the formation of WH, with higher ion concentrations favouring the formation of WH over HAP through destabilisation of the HAP lattice^21–23^. Zinc levels in the prostate peripheral zone are 10–20 times higher than in other normal soft tissues throughout the body, with studies showing that zinc levels decrease with increasing prostate cancer tumour grade (measured by Gleason score or grade group)^24–28^. It would therefore be expected that calcifications in control or low-grade tissue have more WH, similar to some studies in breast calcifications, where WH presence has been linked to benign tissue^6,17,18,29^. Acidification of the tumour microenvironment is also thought to impact the stability of HAP and WH phases, as WH is more stable than HAP in acidic conditions^16,21,30,31^. Therefore, tissue pH and zinc concentration may have opposing effects on WH formation, causing an overall lack of difference between grade groups.

Zinc substitution in HAP and WH crystals can also impact lattice parameters. However, while the tissue zinc levels were found to decrease with increasing grade group, the same was not true for calcifications. There were also no significant correlations between HAP ‘a’ and ‘c’ axes and zinc levels in calcifications, suggesting other factors may be implicated in calcification formation and crystallography. For example, a decreasing level of carbonate substitution into the phosphate site of the HAP lattice would demonstrate similar changes in the ‘a’ and ‘c’ axes. Levels of carbonate and other light elements such as sodium that may impact lattice parameters did not form part of this study but should be explored in future work to establish a comprehensive model of prostate calcification chemistry.

Citrate is also known to interact with HAP crystals in the body, with higher citrate levels thought to decrease crystallinity^32^. The observed differences in HAP coherence lengths may suggest an interaction between citrate and HAP in prostate calcifications. However, the patterns of coherence length shown here suggest it is likely several factors are contributing to differences observed in crystallinity. Citrate levels could not be measured directly in this study; however, this may be a possible avenue for exploration in the future, using techniques such as Raman spectroscopy.

Despite overall correlations with grade group being apparent for some calcification characteristics (HAP ‘a’ and ‘c’ axes, WH ‘a’ axis), grade groups 4 and 5 do not always conform to this pattern. Given that trends are also observed when grouping data by pathological stage, often in opposing directions to grade group, data may be skewed dependent on the distribution of both pathological stage and grade group.

This study has also utilised both grade group and pathological stage to compare crystallographic and elemental parameters of calcifications. This revealed similar correlations between some of these parameters, such as HAP and WH ‘a’ axes, but differing correlations in others. For example, HAP ‘c’ axis has opposing trends when using grade group and pathological stage, and CL002 shows a clear increase with pathological stage while there is no difference with grade group. These findings suggest that the cell morphology (determined by grade group) and extent of a tumour (pathological stage) are reflected in the structure of calcifications in different ways.

## Conclusions, limitations and future outlook

This novel study has started to demonstrate the prognostic potential of the chemistry and crystallography of calcium phosphate calcifications beyond the remit of breast studies. This initial evaluation of calcifications has provided an insight into potentially clinically significant features, evidenced by trends in crystallographic and elemental parameters with grade group and pathological stage. These findings will form the basis of future investigations aiming to differentiate inconsequential disease from consequential disease, offering additional information about the tumour microenvironment.

Some correlations between lattice parameters (‘a’ and ‘c’ axes) and relative levels of zinc have been observed. However, these are not strong associations, and in some cases have the opposite trend to what is expected. Therefore, lattice parameters changes cannot be wholly attributed to the decreasing tissue zinc concentration with increasing grade group and further interrogation of the elemental composition is needed. The use of FFPE tissue also limits the conclusions that can be drawn regarding elemental composition of soft tissue, as the fixation and embedding process has previously been noted to redistribute some elements within the soft tissue. This is less likely in mineral deposits due to elements being bound in the crystal lattice. Future investigation of these deposits with additional analytical techniques such as spectroscopy and light element analysis may reveal further information into ion substitutions (such as carbonate or sodium), as well as providing sufficient data to develop models of calcification formation in the prostate.

Long term, calcification crystallography could be used in prognostic capacity to inform clinicians and patients when considering treatment options by offering an additional tool to stratify patient risk.

## Materials and methods

### Samples

All samples were from radical prostatectomy surgeries, sourced from the Human Biomaterials Research Centre (HBRC) in Birmingham, with ethical approval received through NHS REC (20/NW/0001).

101 formalin fixed paraffin embedded (FFPE) megablocks were scanned using micro computed tomography on a MI-Labs UCT operating at 55 kV and 0.17 mA in ultrafocus mode. 360 projections were collected measuring for 75 ms per point. Data were reconstructed using MI-Labs Recon software with a 15 µm voxel size. The number of calcifications per unit volume were calculated following image thresholding using Fiji (ImageJ) and application of the 3D Objects counter function^33,34^ (Table 1).

**Table 1:** Number of samples per grade group and calcification prevalence in each group based on micro-computed tomographs of megablocks.

Tissue grade	Number of megablocks	Number of blocks containing calcification
Control (adjacent normal tissue)	12	12 (100%)
Grade Group 1 (3 + 3)	12	12 (100%)
Grade Group 2 (3 + 4)	24	23 (96%)
Grade Group 3 (4 + 3)	23	20 (87%)
Grade Group 4 (4 + 4)	8	8 (100%)
Grade Group 5 (4 + 5) & (5 + 5)	22	20 (91%)
Total	101	95 (94%)

A selection of these samples were utilised for X-ray diffraction and fluorescence measurements for the remainder of the study (outlined in detail in Supplementary Tables S1 & S2).

FFPE sections of 10 µm thickness were taken from prostate megablocks across a range of Grade Groups (1–5) and control (adjacent normal tissue from patients with prostate cancer) samples. Control samples were taken from patients with grade group 2 (2 patients) and grade group 5 (2 patients). For clarity, patient PSA scores have been provided for the normal adjacent tissue samples (Supplementary Table S1) with a score of 9 reported for this group. However, the tissue was considered normal via histopathology.

188 calcifications across 49 sections were selected for analysis. Patient age, prostate weight, the volume of prostate occupied by tumour and PSA score were not found to significantly differ between grade groups (Kruskal Wallis, α = 0.05) (Supplementary Table S1). These variables were also analysed using Kendall’s tau correlation coefficient against calcification parameters with all significant (CL030, CL0210, CL220 whitlockite weight %) comparisons with tumour volume found to have weak positive correlations and PSA found to have weak negative correlations (Supplementary Table S2). These variables did not differ significantly by grade or stage; therefore, these parameters were not considered as confounding variables in the analyses. Selection criteria included location in the peripheral zone in the first instance, as most prostate cancer is located here, though calcifications in other zones were also measured for comparison (Supplementary Table S3).

### X-ray diffraction

Data were collected on the i18 beamline at Diamond Light Source, Didcot UK, with a beam energy of 12 keV, spot size of 10 × 10 μm and an Excalibur detector, with samples mounted normal to the X-ray beam. µCT images of sections and a microscope mounted at 45° to the sample stage were used to identify regions of interest and the presence of calcium was confirmed using X-ray fluorescence at 12 keV using a Vortex Silicon Drift Detector. Individual calcifications were interrogated using XRD with lines across the longest axis or maps of whole calcifications, using a step size of 10 µm and collection time of 15 s per point.

2-dimensional data was azimuthally integrated into 1-dimensional data following application of a detector mask and calibration with a silicon standard using the Diamond Analysis WorkbeNch (DAWN) software (V2.26.0, Diamond Light Source)^35^. Phase identification was conducted using the International Centre for Diffraction Data (ICDD) database (PDF-5+, 2023) and microstructural analysis was carried out using JADE Pro (ICDD).

Whole pattern analysis was utilised to fit space groups P6_3_/m and R3c to the HAP and WH phases respectively This refinement involves a least squares approach to fit a predefined pattern to the presented data, permitting the refinement of relative weights and physical dimensions of each mineral phase. Lattice parameters (‘a’ axis and ‘c’ axis) are two of the physical dimensions of the individual units of crystals in a lattice that define their geometry. These parameters are highlighted in Gosling et al.^16^. ‘a’ and ‘c’ axis lengths are affected by ionic substitutions, including carbonate, sodium, magnesium and zinc. For example, zinc or magnesium ion substitution into HAP will cause a decrease in the ‘c’ axis, whereas sodium ion substitution will cause an increase in the ‘c’ axis^36–38^. In reality, there will be multiple substitutions occurring in biological minerals, therefore these measures can only give an indication of these substitutions and not definitive causative correlations.

Individual peaks (Bragg maxima) for HAP (002, 030) and WH (0210, 220) were analysed to determine coherence length as these are non-overlapped peaks for nanocrystalline HAP and WH respectively. Coherence length, CL, (or domain size) is a measure of crystallinity which represents the average distance within a crystal over which lattice order persists. CL can be quantified for any particular Bragg peak using the Scherrer equation:

$$CL = \frac{K\lambda }{{\beta_{hkl} cos\theta_{hkl} }}$$

where K is the shape factor (0.9), λ is the wavelength (0.1033 nm), β_hkl_ is the FWHM (assuming no instrumental broadening from the synchrotron instrumentation) and θ is the Bragg angle.

### X-ray fluorescence

X-ray fluorescence maps were collected at 12 keV using a spot size of 10 × 10 µm, a 1 s collection time and a Vortex silicon drift detector. Elemental assignment and peak intensities of calcium, zinc and iron were measured using PyMCA^39^, and values calculated relative to total ion content for the tissue and calcification portions of the tissue sections.

### Data grouping

Data were separated by prostatic zone for further analysis due to the difference in frequency of cancer in each zone and tissue microenvironments. The PZ forms the majority of prostatic glandular tissue and has the highest frequency of prostate cancer (70–80%); the TZ surrounds the urethra and increases in size throughout life, representing most benign prostatic hyperplasia and 20% of cancers; and the CZ surrounds the ejaculatory ducts and accounts for < 5% of prostate cancers^13,40^. The FMZ consists of smooth muscle bundles which cover the anterior surface of the prostate, but prostate cancer originating from this zone is rare.

Prostatic zones CZ and TZ were grouped together as it was not possible to confidently separate these zones using the µCT images collected before sectioning. PZ calcification data were separated into control (adjacent normal tissue) vs. malignant, however, the relative numbers for these groups were uneven, where n = 2 and n = 69 for control and malignant, respectively. PZ calcifications were further analysed using the International Society of Urological Pathology (ISUP) Grade (Groups 1–5), which is a classification system for urological tumours, specifically for prostate cancer^41^. Data were also separated by pathological stage information (T2, T3a, T3b, T4), which describes the size and area of the cancer^42^. Clinically, patient risk is stratified by combining PSA score, ISUP Grade Group and pathological stage, in a tool called the Cambridge Prognostic Group, however as PSA information was not available for all samples, this approach was considered inappropriate for this study.

### Statistical analysis

All included parameters failed a Shapiro–Wilk test for normality, therefore median values per calcification were calculated before carrying out further analysis averaging by prostatic zone and tissue grade. Kruskal–Wallis tests were utilised to compare groups, with a Dunn-Sidak post-hoc correction for multiple comparisons. All quoted *p* values are Dunn-Sidak corrected values unless otherwise stated.

Kendall’s tau correlation coefficients (r_τ_) were calculated and reported alongside corresponding p values. A weak correlation is reported for |r_τ_|< 0.4, a moderate correlation is reported for 0.4 <|r_τ_|< 0.8 and a strong correlation is reported for |r_τ_|> 0.8. Only values with *p* < 0.05 are reported in the text, however all r_τ_ and *p* values are provided in the Supplementary Information for completeness. Kendall’s tau was deemed the most appropriate statistical test in this case due to the relatively small sample numbers and non-parametric nature of the data. Kendall’s tau is also appropriate to use with ordinal data (grade group and pathological stage), therefore is used throughout the paper.

All mathematical analyses were performed using MATLAB 2023a (The MathWorks Inc., Natick, Massachusetts).

## Supplementary Information

Supplementary Information.

## Data Availability

The data that support the findings of this study are available from the corresponding author upon reasonable request.
