# Supplementary material for: Prostate microcalcification crystallography as a marker of pathology
Source: Sci Rep. 2025 Apr 29;15:14979. doi: 10.1038/s41598-025-98692-8 (PMC12041448; doi:10.1038/s41598-025-98692-8)
Supplement: Supplementary file 1 — Supplementary Information. [file 41598_2025_98692_MOESM1_ESM.pdf]

Prostate microcalcification crystallography as a marker of pathology

Sarah B. Gosling, Emily L. Arnold, Lois Adams, Paul Cool, Kalotina Geraki, Mark Kitchen, Iain D. Lyburn, Keith D. Rogers, Tim Snow, Nicholas Stone & Charlene E. Greenwood

Supplementary Information

Title	Content	Page
Table S1	Patient and histological information for histological sections cut for XRD analysis.	2
Table S2	Kendall's tau correlation coefficients between tumour volume, patient age, PSA and prostate weight and crystallographic parameters in PZ calcifications.	3
Table S3	Calcification zonal and T stage information for histological sections cut for XRD analysis.	4
Table S4	Kendall's tau correlation coefficients between Zn ratios in calcifications and the surrounding tissue and calcification parameters	5
Figure S1	Mineral phase, crystallographic and elemental parameters of calcifications split by zone.	6 – 7
Figure S2	Mineral phase, crystallographic and elemental parameters of calcifications in control and malignant tissue.	8 – 9

Supplementary Table S1: Patient and histological information for histological sections cut for XRD analysis. Mean values and standard deviation are presented for each variable. *Control samples are from adjacent normal tissue in prostate cancer patients; therefore this control sample has an elevated PSA score associated with the patients prostate cancer.

Grade Group	Number of sections	Patient age / years	Prostate mass / g	Volume of prostate occupied by tumour / %	Samples with PSA information	PSA / ng / ml
Control (adjacent normal)	4 (8 %)	61.0 ± 10.7	58.8 ± 27.7	4.3 ± 2.8	1 (25 %)	9.0 ± 0*
Grade Group 1 (3+3)	8 (16 %)	59.8 ± 7.6	57.1 ± 23.3	13.0 ± 8.5	5 (63 %)	9.9 ± 7.5
Grade Group 2 (3+4)	8 (16 %)	61.5 ± 9.4	60.0 ± 18.4	17.7 ± 13.5	5 (63 %)	7.6 ± 4.6
Grade Group 3 (4+3)	12 (24 %)	65.2 ± 6.8	44.1 ± 14.7	19.8 ± 16.1	11 (92 %)	11.1 ± 6.9
Grade Group 4 (4+4)	5 (10 %)	65.6 ± 5.2	48.3 ± 9.3	31.8 ± 46.3	1 (20 %)	10.0 ± 0
Grade Group 5 (4+5) & (5+5)	12 (24 %)	65.8 ± 6.4	38.3 ± 11.6	37.5 ± 32.2	2 (17 %)	20.1 ± 14.0
Average		63.5 ± 7.5	48.9 ± 18.4	22.1 ± 24.0	25 (51 %)	10.7 ± 7.1
p	-	0.491	0.052	0.152	-	0.610

Supplementary Table S2: Kendall's tau correlation values (r_τ) with p values between tumour volume, patient age, PSA and prostate weight and crystallographic parameters in PZ calcifications including ('a' and 'c' axes) and coherence lengths (CL) for hydroxyapatite (HAP) and whitlockite (WH) and WH weight percentage. p values highlighted in bold indicate a 95 % or higher level of significance.

**Calcification parameter**	**Tumour volume**		**Age**		**PSA**		**Prostate Weight**	
	r_τ	*p*	r_τ	*p*	r_τ	*p*	r_τ	*p*
CL002 (HAP)	0.0820	*0.3361*	0.0760	*0.3743*	-0.0619	*0.5247*	-0.0538	*0.5538*
CL030 (HAP)	0.2370	***0.0053***	0.0439	*0.6086*	-0.1959	***0.0424***	-0.1263	*0.1617*
CL0210 (WH)	0.2591	***0.0023***	0.1600	*0.0605*	-0.2178	***0.0239***	-0.0005	*1.0000*
CL220 (WH)	0.0960	*0.2597*	0.0772	*0.3662*	-0.2247	***0.0198***	-0.0607	*0.5030*
Whitlockite wt. %	0.1917	***0.0412***	0.0652	*0.4905*	-0.3086	***0.0036***	-0.0089	*0.9344*
HAP 'a' axis	-0.0944	*0.2780*	-0.2549	***0.0034***	0.1004	*0.3053*	-0.1668	*0.0693*
HAP 'c' axis	-0.1279	*0.1371*	0.2759	***0.0013***	-0.1034	*0.2868*	0.1207	*0.1854*
WH 'a' axis	-0.1700	*0.2351*	-0.1534	*0.2809*	0.1262	*0.4368*	-0.1691	*0.2795*
WH 'c' axis	-0.1637	*0.2517*	0.1750	*0.2160*	-0.0669	*0.6879*	0.2293	*0.1383*

Supplementary Table S3: Calcification zonal and T stage information split by grade group for histological sections cut for XRD and XRF analysis. Percentages represent the relative distribution of calcifications in each zone/stage for each tissue grade group. FMZ: fibromuscular zone, PZ: peripheral zone, CZ: central zone, TZ: transition zone.

Grade Group	Number of calcifications measured	Calcifications measured per section	Zone			T stage			
			FMZ	PZ	CZ/TZ	T2	T3a	T3b	T4
Control	10	2.50	0	2 (20 %)	8 (80 %)	10 (100 %)	0	0	0
Grade Group 1 (3+3)	25	3.13	1 (4 %)	18 (72 %)	6 (24 %)	11 (44 %)	0	14 (56 %)	0
Grade Group 2 (3+4)	26	3.25	1 (4 %)	10 (38 %)	15 (58 %)	19 (73 %)	7 (27 %)	0	0
Grade Group 3 (4+3)	42	3.50	4 (10 %)	8 (19 %)	30 (71 %)	30 (72 %)	11 (26 %)	1 (2 %)	0
Grade Group 4 (4+4)	33	6.60	4 (12 %)	14 (42 %)	15 (45 %)	0	28 (85 %)	0	5 (15 %)
Grade Group 5 (4+5) & (5+5)	52	4.33	2 (4 %)	19 (37 %)	31 (60 %)	7 (14 %)	10 (19 %)	35 (67 %)	0
Total	188	3.84	12 (6 %)	71 (38 %)	105 (56 %)	77 (41 %)	56 (30 %)	50 (26 %)	5 (3 %)

Supplementary Table S4: Kendall's tau correlation coefficients (r) with p values between Zn ratios in calcifications and the surrounding tissue and lattice parameters ('a' and 'c' axes) and coherence lengths (CL) for hydroxyapatite (HAP) and whitlockite (WH) and WH weight percentage. p values highlighted in bold indicate a 95 % or higher level of significance.

			**HAP 'a' axis**	**HAP 'c' axis**	**WH 'a' axis**	**WH 'c' axis**	**CL002**	**CL030**	**CL0210**	**CL220**	**WH wt. %**
Zn ratio in calcifications	All Data	r_τ	0.104	-0.090	-0.005	0.196	-0.085	0.065	-0.071	-0.061	-0.032
		p	*0.072*	*0.119*	*0.966*	***0.033***	*0.127*	*0.240*	*0.199*	*0.270*	*0.601*
	PZ Data	r_τ	0.051	-0.017	0.181	0.041	-0.035	0.095	-0.247	-0.231	-0.140
		p	*0.624*	*0.876*	*0.298*	*0.836*	*0.636*	*0.194*	***0.012***	***0.019***	*0.201*
	CZ/TZ Data	r_τ	0.172	-0.124	-0.182	0.068	0.098	-0.151	0.029	-0.004	-0.011
		p	***0.023***	*0.106*	*0.142*	*0.590*	*0.336*	*0.138*	*0.692*	*0.959*	*0.896*
Zn ratio in surrounding tissue	All Data	r_τ	-0.133	0.035	-0.101	-0.037	-0.026	-0.080	0.011	-0.016	-0.138
		p	***0.023***	*0.556*	*0.279*	*0.695*	*0.796*	*0.425*	*0.848*	*0.775*	***0.028***
	PZ Data	r_τ	-0.240	0.108	0.111	0.007	-0.023	0.024	-0.125	-0.040	-0.308
		p	***0.022***	*0.298*	*0.550*	*1.000*	*0.687*	*0.678*	*0.220*	*0.702*	***0.006***
	CZ/TZ Data	r_τ	-0.031	-0.075	-0.220	-0.091	-0.066	0.098	0.052	0.007	-0.081
		p	*0.685*	*0.335*	*0.075*	*0.469*	*0.374*	*0.185*	*0.483*	*0.924*	*0.322*

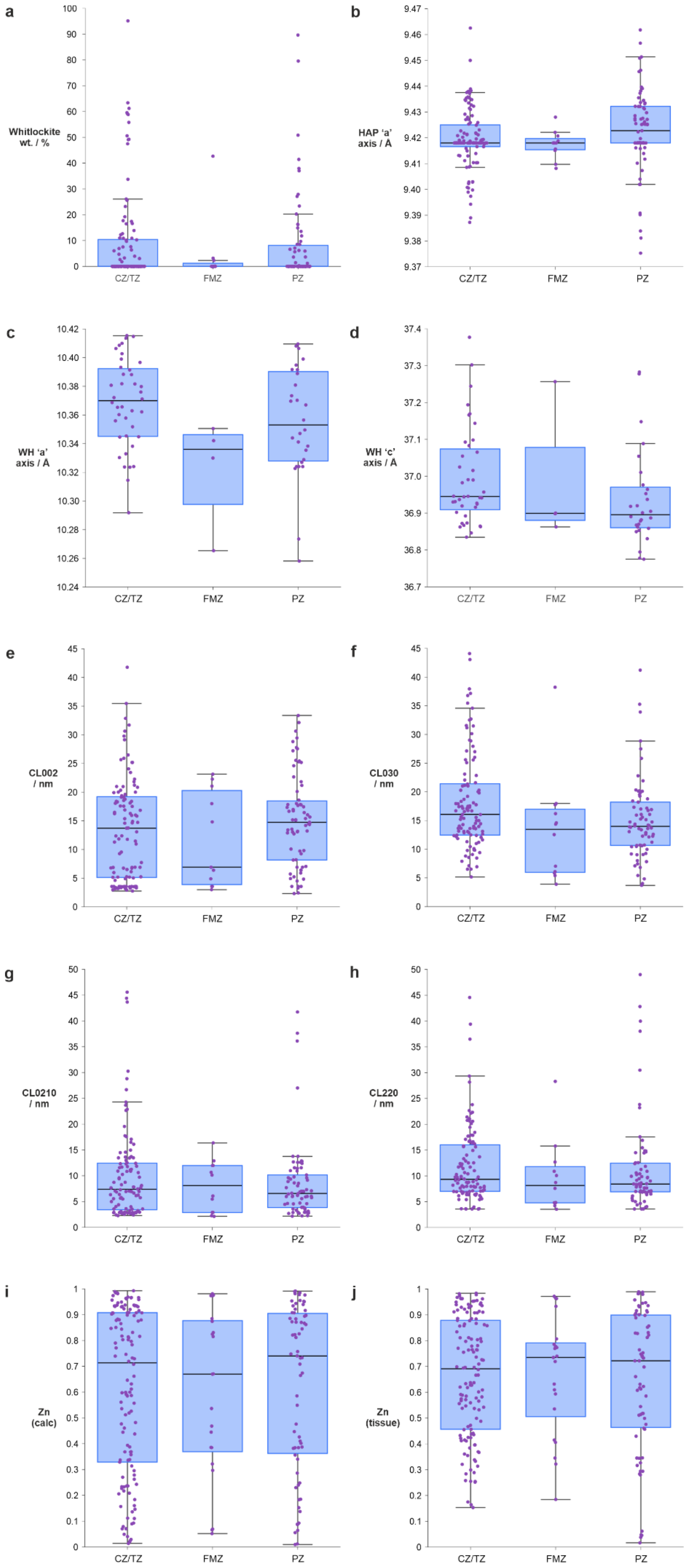

a
Whitlockite wt. / %
b
HAP 'a' axis / Å
c
WH 'a' axis / Å
d
WH 'c' axis / Å
e
CL002 / nm
f
CL030 / nm
g
CL0210 / nm
h
CL220 / nm
i
Zn (calc)
j
Zn (tissue)
CZ/TZ
FMZ
PZ

Supplementary Figure S1: Mineral phase, crystallographic and elemental parameters of calcifications split by zone. a) WH weight percentage. b) 'a' axis value for HAP, c & d) 'a' and 'c' axis values for WH. e & f) CL measured along 002 and 030 for HAP, g & h) CL measured along 0210 and 220 for WH. i & j) Zinc calculated as a percentage of total metal ion content (calcium, zinc and iron) for calcifications and tissue.

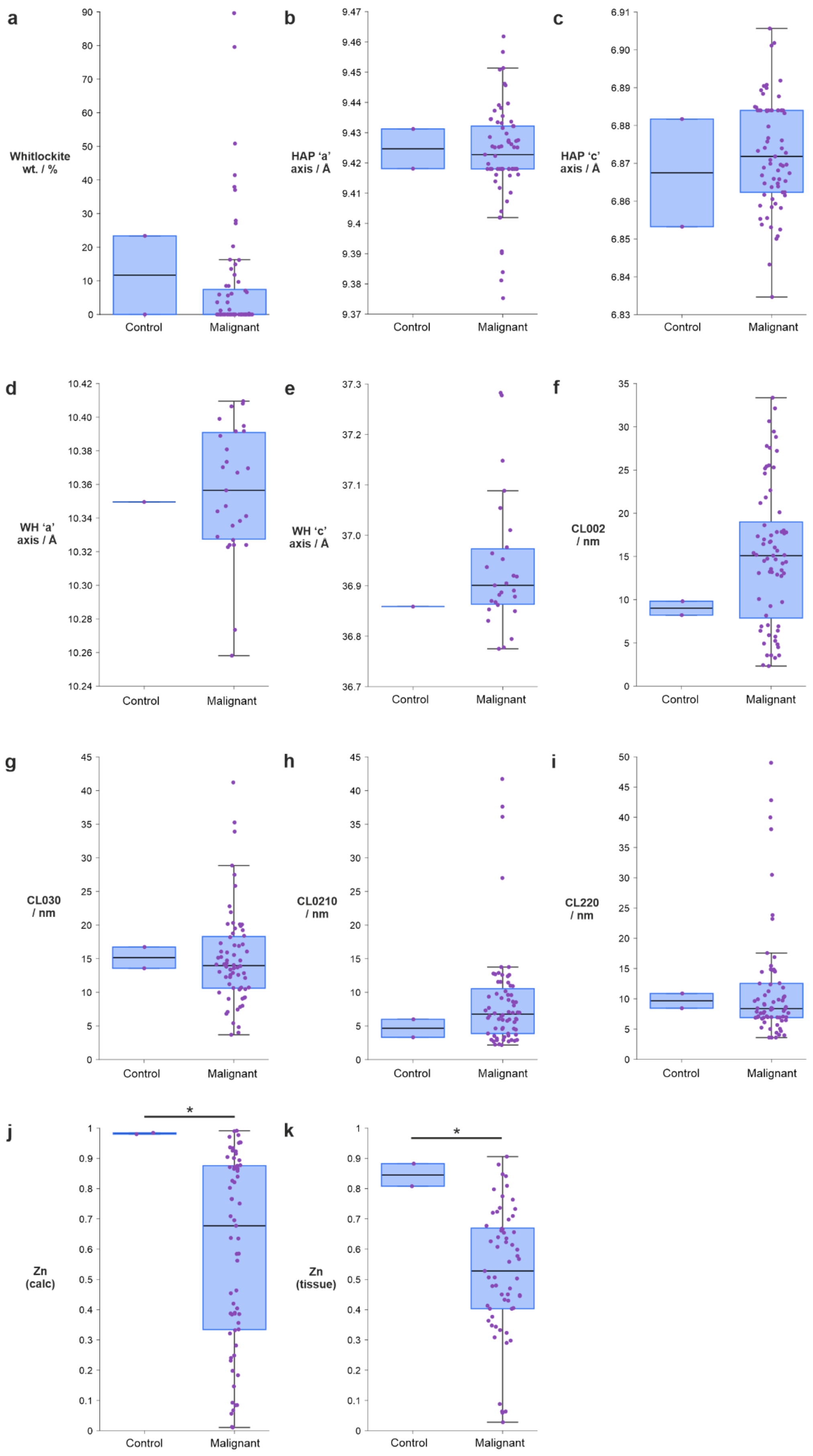
a
Whitlockite wt. / %
b
HAP 'a' axis / Å
c
HAP 'c' axis / Å
d
WH 'a' axis / Å
e
WH 'c' axis / Å
f
CL002 / nm
g
CL030 / nm
h
CL0210 / nm
i
CL220 / nm
j
Zn (calc)
k
Zn (tissue)
Control
Malignant
*

Supplementary Figure S2: Mineral phase, crystallographic and elemental parameters of calcifications in control and malignant tissue. a) WH weight percentage, b – e) lattice parameters for HAP (b & c) and WH (d & e), f & g) CL measured along 002 and 030 for HAP, h & i) CL measured along 0210 and 220 for WH, j & k) Zinc calculated as a percentage of total metal ion content (calcium, zinc and iron) for calcifications and tissue. *$p<0.05$.
